# Letting Go of the Negative, Holding on to the Positive? Within-Person Trajectories of Affective Habituation to Negative and Positive Stimuli

**DOI:** 10.1177/01461672251348486

**Published:** 2025-08-03

**Authors:** Elizabeth Yartsev, Oliver P. John, Özlem N. Ayduk

**Affiliations:** 1 University of California, Berkeley, CA, USA

**Keywords:** affective habituation, emotion, anxiety, information processing biases

## Abstract

The *differential affective habituation* hypothesis argues that affective reactions decrease faster for positive than for negative information due to the greater evolutionary importance of threat over reward. However, limited evidence and low-powered studies hinder strong conclusions about this hypothesis from the extant literature, a gap the present research aimed to address. Because anxiety entails heightened threat anticipation, a second aim was to examine if higher anxiety intensifies this differential habituation pattern (i.e., *anxiety potentiation hypothesis*). Two experiments (*N*_1_ = 104, *N*_2_ = 211) provided within-subject exposure to International Affective Picture System (IAPS) images, manipulated stimulus valence, and assessed anxiety at baseline (Studies 1 and 2) or following an anxiety manipulation (Study 2). Results supported both hypotheses, highlighting the importance of examining positive habituation as a key mechanism in psychological functioning and suggesting that, despite its potential survival function, differential habituation may carry psychological costs.

In everyday life we are repeatedly exposed to a variety of emotional triggers, both positive and negative in nature, some internally generated and others initiated by external situations. These emotional triggers elicit a rich variety of emotional reactions that tend to linger with us for a certain period of time before they fade away and other emotions take center stage. However, emotions are unlikely to dissipate all at the same pace; some emotions might fade away relatively quickly, whereas others may linger on for much longer.

Researchers interested in the process of hedonic adaptation have examined how emotions elicited by major life events lose their potency within extended time frames such as weeks, months, and even years (e.g., Lyubomirsky, 2011). However, we know relatively less about how immediate affective reactions triggered by momentary situational factors change dynamically over short periods of time, and how such trajectories might differ as a function of stimulus valence. Therefore, one of the goals of the current study was to examine whether *affective habituation*—the process by which emotional reactions lose their intensity when an eliciting stimulus is repeatedly presented (Averill et al., 1972), might be different for positive versus negative emotional triggers.

There is extensive research showing that humans are equipped with cognitive, affective, and behavioral response systems that give less weight to reward than to threat, presumably because the former is less relevant to survival (Rozin & Royzman, 2001). Extending this reasoning to how people’s emotional reactions to a stimulus might change with repeated exposure over time, we hypothesized that habituation should occur at a faster rate for positive than negative stimuli—a prediction we refer to as the *differential affective habituation hypothesis*. Although the extant literature provides some support of this expectation, methodological issues prevent us from being able to draw strong inferences about how habituation is modulated by stimulus valence. Providing a strong test of the differential affective habituation hypothesis was, thus, the primary goal of the present research.

A second goal was to examine if the expected pattern of differential habituation gets further potentiated by anxiety (anxiety potentiation hypothesis). Under threat (whether perceived or actual), positive information might be deemed even less relevant to survival while negative information becomes all the more important. Since anxiety entails a heightened sense of threat (e.g., Beck & Clark, 1997; Grupe & Nitschke, 2013), people higher in anxiety might thus habituate even faster to positive and/or even slower to negative information—a possibility that led us to test if anxiety symptomatology moderates the effect of stimulus valence on affective habituation. Below, we elaborate on the reasoning for both of these hypotheses.

## Affective Habituation

Affective habituation refers to the process of experiencing a progressive decrease in physiological, emotional, and behavioral responses to a repeatedly presented stimulus over a relatively short period of time (Averill et al., 1972; Thompson & Spencer, 1966). The phenomenon of habituation has been described across a wide range of species, from simple organisms to humans, and is believed to serve as a pre-requisite for more complex forms of learning, memory, and cognition, due to its role in filtering out irrelevant information while processing a wide range of stimuli in the environment (e.g., Rankin et al., 2009).

Affective habituation in humans is typically studied in laboratory studies where an affectively charged stimulus is presented repeatedly, and individuals’ emotional responses to the stimulus are tracked across time (e.g., Bradley et al., 1993; Leventhal et al., 2007). In other words, this concept of habituation focuses on trial-by-trial changes in emotional reactions to repeated stimuli presentation.

The relatively small literature on affective habituation that exists to date has focused primarily on negative stimuli (i.e., *negative habituation*) because it originated in research on systematic desensitization (e.g., Foa & Kozak, 1986)—a therapeutic technique for treating phobia that centers on the assumption that repeated exposure to fear stimuli of progressively increasing intensity leads to significant reductions in fear arousal and extinction of phobic behavior (e.g., see Foa & McLean, 2016, and Hofmann, 2008 for reviews). Indeed, the empirical literature on exposure therapy has established that emotional reactions to negative stimuli that are repeatedly presented lose their intensity over time.

In contrast, studies on positive habituation are much further and fewer in between despite the relevance of positive affect for psychological functioning (see Fredrickson & Losada, 2005 for review). Table 1 presents the handful of empirical papers (*k* = 6) we have been able to identify as focusing specifically on affective habituation to positive stimuli, which altogether suggest three important takeaways. First, there is consistent evidence showing that with repeated exposure, emotional responses to positively valenced stimuli, too, lose their intensity over time. For example, prior subliminal exposure to extremely positive (and negative) words reduces their emotional impact during subsequent supraliminal exposure (Dijksterhuis & Smith, 2002). Similarly, emotional reactions to positive images decay linearly over time with repeated exposure (Leventhal et al., 2007; Mantua & Ready, 2019).

**Table 1.** Summary of Empirical Studies on Habituation to Positive Stimuli.

Article	Population and sample size	Stimuli valence and (manipulation type)	Type of stimuli
Leventhal et al. (2007) (Studies 1 & 2)	Undergraduates *N*_1_ = 90 *N*_2_ = 107	Positive only	IAPS images
	Key finding(s): *Significant decrease in intensity of positive affect with repeated exposure.*		
Mantua and Ready (2019)	Young adults with and without brain injury *N* = 45/group	Positive and negative (within-subjects)	IAPS images
	Key findings: *Significant decrease in affect with repeated exposure to positive but not negative images. Valence×Repetition interaction was statistically significant in the whole sample.*		
Dijksterhuis and Smith (2002) Study 1	Undergraduates *N* = 37	Positive and negative (within-subjects)	Affectively charged words with extreme intensity
	*Key findings: Significant reduction in evaluative intensity to supraliminally presented positive and negative words following prior subliminal exposure. Valence×Repetition interaction was not statistically significant.*		
Dijksterhuis and Smith (2002) Study 2	Undergraduates *N* = 16	Positive and negative (within-subjects)	Affectively charged words with extreme intensity
	*Key findings: Significantly longer reaction times to previously seen positive and negative words than to not previously seen ones (on good-bad evaluations in a standard IAT task) indicating exposure led affectively charged words to lose their subjective extremity. Valence × Repetition interaction was not statistically significant.*		
Wright et al. (2001)	Healthy right-handed men from the community *N* = 8	Positive and negative (within-subjects)	Images of happy versus fearful facial expressions
	*Key findings: Significantly greater reduction in left dorsolateral prefrontal cortex activation to happy versus fear faces with repeated exposure. Valence × repetition interaction was significant. Activation in the right amygdala and left premotor cortex declined significantly with exposure but Valence × Repetition interaction was not statistically significant.*		
Bradley et al. (1993)	Undergraduates *N* = 17	Positive and negative (within-subjects)	IAPS images
	*Key findings: Significant decline in the magnitude of the startle response with repeated exposure to positive and negative images. Valence × Repetition interaction was not statistically significant.*		

IAPS = International Affective Picture System.

Second, there are just a few studies that have included *both* positive and negative stimuli using within-subjects designs and are therefore able to speak directly to the differential habituation hypothesis. Furthermore, many if not all of the relevant studies have relied on very small sample sizes, leaving questions about power and replicability to be addressed.

Third, the extant evidence for the *differential habituation* hypothesis remains inconclusive. Supportive evidence is presented by Mantua and Ready (2019) who found that, over repeated exposure, positive images were rated lower in intensity and negative images were not. Most critically, the Valence × Repetition interaction—the critical statistic that directly tests the differential habituation hypothesis was statistically significant. However, none of the other studies reviewed in Table 1 yielded a significant interaction, suggesting that either the *differential habituation* hypothesis is wrong or the studies were so underpowered that they could not detect an interaction effect.

Findings from seemingly similar literatures do not provide direct evidence for the differential habituation hypothesis either. For example, almost all studies on hedonic adaptation use between-subject designs (e.g., comparing lottery winners to paralyzed accident victims), and unlike habituation, track changes in people’s well-being to a single extreme life event (getting paralyzed) over extended periods of time, such as months or years (e.g., Lyubomirsky, 2011). Similarly, affective chronometry studies, which examine how quickly emotion peaks following stimulus onset, or how quickly it returns to baseline after the peak, are only able to provide indirect insights into habituation (e.g., Hemenover, 2003).

Finally, although the literature on hedonic decline (e.g., Galak & Redden, 2018) examines specific instances of habituation, including sensory-specific satiety (e.g., how the intensity of our positive reactions to sensory stimuli, such as food, declines over time as we become accustomed to them), it focuses primarily on the decline in pleasure or reward value of positive sensory stimuli. Furthermore, within-person comparisons of hedonic decline across stimuli that differ in valence (e.g., liked vs. disliked foods), which are key to the differential habituation hypothesis, are relatively rare (cf. Schellenberg et al., 2008).

Taken altogether, the current knowledge on the effect of valence on affective habituation either comes from a relatively small number of underpowered studies, which have yielded mixed results or from adjacent literatures that do not address habituation per se. Therefore, one of the main aims of the present study was to contribute to this body of work by testing whether affective habituation is moderated by stimulus valence in a well-powered study using a within-subjects design.

## The Anxiety Potentiation Hypothesis: The Role of Anxiety in Affective Habituation

The differential affective habituation hypothesis examines normative differences in the rates of habituation to positive versus negative stimuli. However, factors such as heightened anxiety (whether due to trait, or state/situational differences) may influence these normative trajectories. Specifically, because heightened anxiety is characterized by an increased anticipation of potential threat (e.g., Beck & Clark, 1997; Grupe & Nitschke, 2013), it may shift the relative emphasis on threat versus reward at a given moment, modulating how individuals habituate to both positive and negative stimuli.

Some, though not all, of the existing evidence is consistent with this expectation showing for example that people higher in anxiety display reduced habituation of physiological reactions under threat (Chattopadhyay et al., 1980; Eckman & Shean, 1997). Furthermore, valence-based information processing biases, including hyper-vigilance for threat, inability to disengage from negative cues, biased interpretation of ambiguous cues as threat, and perseverance on negative content, and disruptions in aversive prediction error signaling, are well-established in the etiology and maintenance of anxiety disorders (see Bar-Haim et al., 2007 for review).

With respect to positive stimuli, attention and memory biases in processing positive information are also common in social anxiety (see Frewen et al., 2008, for meta-analyses) including greater inattention (Taylor et al., 2011) and faster disengagement from positive social information (Chen et al., 2012), as well as a more negatively biased recall of positive feedback (Glazier & Alden, 2017), and deficits in reward learning (Morris et al., 2015). Some studies have also demonstrated positive affect deficits in social anxiety (Eisner et al., 2009; Kashdan, 2007).

When positive and negative information processing biases associated with anxiety are taken together, they suggest that people higher in anxiety might deem positive information even less relevant to survival while judging negative information to be even more important. The implication of this possibility for our purposes is that the normative pattern of differential habituation we hypothesized in the preceding section might be particularly potentiated at higher levels of anxiety. To the best of our knowledge, however, there is very little data that directly addresses this question. Moreover, basic science research in non-clinical populations often examine the link between anxiety and negative habituation but is mostly silent on positive habituation (e.g., Chattopadhyay et al., 1980; Eckman & Shean, 1997). Finally, many of the studies reviewed in Table 1 did not assess anxiety or related constructs, and those that did yielded a mixed pattern of findings. Specifically, Mantua and Ready (2019) found neither positive nor negative habituation to be significantly related to anxiety symptomatology. Therefore, our second goal was to test the anxiety potentiation hypothesis that the expected pattern of differential habituation should be further potentiated at higher levels of anxiety.

## Overview of the Present Studies and Hypotheses

We conducted two experiments to test differential habituation hypothesis and examine its relationship to anxiety symptomatology (anxiety potentiation hypothesis) in non-clinical populations. Study 1 repeatedly presented participants with images that were ideographically selected to elicit the most intense positive and negative reactions. Study 2 pre-registered our methods and hypotheses, aiming to replicate Study 1 findings using a fixed set of normatively rated positive and negative stimuli. Individual differences in anxiety were measured at the symptom level in both studies as symptomatology (vs. personality trait) measures should better capture dynamic changes in the relative weighing of threat versus reward cues and their impact on affective habituation. Additionally, state anxiety was manipulated in Study 2 to explore the causal effect of situationally induced anxiety on affective habituation.

For the differential habituation hypothesis, we hypothesized repeated exposure would reduce intensity of emotional reactions at a faster rate for positive than negative stimuli (i.e., significant Valence × Repetition interaction). For the anxiety potentiation hypothesis, we hypothesized people higher in anxiety would show an even larger difference in their habituation rates to negative versus positive stimuli (i.e., significant Valence×Repetition×Anxiety interaction).

## Study 1: Testing Differential Habituation with Idiographic Stimuli

### Methods

#### Participants

Our study design incorporated one primary between-subjects variable (anxiety, continuous) and two within-subjects variables (valence and repetition). Following the recommendation to use 30 to 50 participants at each level of a between-subjects variable to approach 80% power (VanVoorhis & Morgan, 2007; Simmons et al., 2013) we treated anxiety as if it is a two-level (high vs. low) between-subjects variable, aiming to recruit 50 participants per cell (i.e., 100 participants total).

Participants were recruited from a paid subject pool managed by a large public university on the West Coast of the United States. Eligibility criteria included being 18 or older, and proficient in English, and having no self-reported past or current post-traumatic stress disorder diagnosis. The final sample (*N* = 105) consisted mostly of students and a few employees with the following demographic characteristics: 63.5% female; average age of 21.5 (Med = 20, *SD* = 4.44); 57.7% Asian-American, 20.2% European-American, 6.7% Latinx, 1.9% African-American, 1% Pacific Islander, 6.7 mixed race, and 5.8% other. One participant was excluded from all data analyses for failing two of the three attention checks in the surveys.

#### Procedure

Participants completed the study individually in a private cubicle in a supervised group testing session (up to 20 participants). The experiment included three phases. To select idiographic stimuli for the habituation task, Phase 1 presented participants with 10 positive, 10 negative, and 6 neutral images from the IAPS (Lang et al., 2008), all depicting either human emotions or human interactions. They were randomly presented, and participants rated their affect after each image. For each individual, the two most negatively and the two most positively rated images were selected by the computer to be used as their idiographic stimuli in the following habituation task.

**Figure 1.**
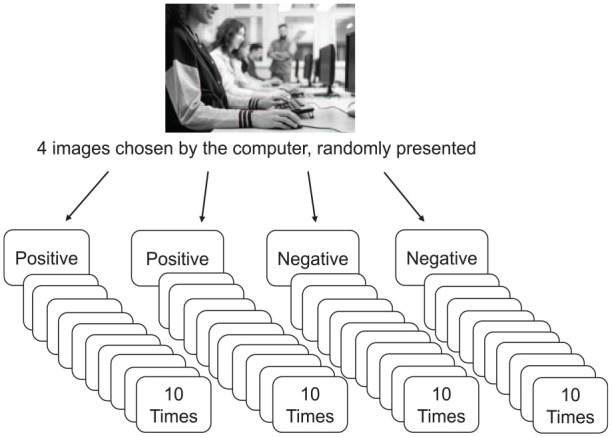
Flowchart of habituation paradigm (Phase 2) of Study 1. *Note.* Following the practice rating task of 26 images, the computer selected the four images rated highest on emotional intensity (two positive and two negative). The four images were presented in random order, in four blocks of 10 repetitions per block. Image element from Young female participant at computer by Ground Picture, 2016, Shutterstock (Asset ID: 464455418). Used under license.

As shown in Figure 1, Phase 2 consisted of the habituation task that included four blocks of presentation, one for each of the four pictures selected in the previous phase (two negative, two positive), presented in random order. Within a block, each picture was presented 10 times for 5 s. Participants were instructed to look at each image presentation for the full time it was presented and to rate their reactions to each presentation within a 5 s response window.

In Phase 3, participants completed a survey designed to assess individual differences in current anxiety symptoms as well as a number of other measures not related to the present research (see Supplementary Materials for a list of additional measures). All study materials, data, and syntax for analyses are accessible via the following link.

##### Habituation Stimuli Selection

Selection of images for Phase 1 was based on valence (1 = *maximum negativity*, 9 = *maximum positivity*) and arousal (1 = *lowest arousal*, 9 = *highest arousal*) norms reported by Ito et al. (1998). The stimuli set included 10 positively valenced (*M* = 7.14, *M_SD_* = 1.8), and 10 negatively valenced (*M* = 2.33, *M_SD_* = 1.6) images, which were matched on arousal (*M*_pos_ = 6.49, *SD*_pos_ = 2.11; *M*_neg_ = 6.19, *SD*_neg_ = 2.44; *t* < 1). An additional six neutral (*M* = 4.6, *M_SD_* = 1.57) images were also included in the pilot phase as distractors.^1^

#### Measures

##### Anxiety Symptomatology

Anxiety symptomatology was measured with three scales all measuring anxiety symptoms during the past week rated on a 4-point rating scale (0 = *never/not at all* to 3 = *always/very much*): (a) the Burns Anxiety Inventory (BAI: Burns, 1989) assesses the experience of 33 anxiety symptoms including anxious feelings, for example, “feeling tense,” anxious thoughts, for example, “having racing thoughts,” and physical symptoms, for example, “trembling” (α = .91; total-sum *M* = 16.46, *SD* = 11.88); (b) the anxiety subscale of the DASS-21 (Lovibond & Lovibond, 1995) measures seven symptoms related to autonomic arousal and subjective experience of anxiety, for example, “ I felt I was close to panic” (α = .76; total-sum *M* = 6.58, *SD* = 6.22); (c) the stress subscale of the DASS-21 consists of seven items measuring nervous or nonspecific arousal and difficulty in relaxing, for example, “I felt that I was using a lot of nervous energy” (α = .83; total-sum *M* = 11.19 and *SD* = 8.11). According to the DASS-21 scoring instructions, the subscale scores were multiplied by 2.

As expected, all three scales correlated highly with each other (all *r*s > .66). We, therefore, computed an overall anxious symptomatology score by averaging the three standardized scale scores (α = .87).^2^

##### Affect During Habituation

Following each image presentation participants were asked “Please rate *how this image makes you feel right now, at this moment*” using a sliding scale (from −3 = “*extremely negative*” to +3 = “*extremely positive*,” with 0 = “*neither positive nor negative*”). They were allowed to rate their affect anywhere on the scale with increments of 0.10 (which were not shown to the participants), which were recorded on a scale from −30 (*extremely negative*) to +30 (*extremely positive*) by the computer. We used this more differentiated scale from −30 to +30 for all our analyses. To align affect ratings to positive and negative stimuli, we multiplied the ratings for negative stimuli by −1. Higher numerical values on this metric (range: 0–30) indicate stronger affective reactions to both negative and positive images. For each image, affect ratings across 10 presentations constituted the key dependent variable in all analyses.

Additionally, affect ratings to the first presentation of each image in Phase 2 were used to operationalize participants’ baseline affect (*M* = 22.39; *SD* = 7.92; range: −6–30). That these ratings (on average) approached the high end of the 0 to 30 rating scale was expected since the experimental stimuli were idiographically selected from Phase 1 to represent the most negative and positive images specific to each participant. However, baseline affect was even stronger for negative (*k* = 2, *M* = 24.8, *SD* = 6.09) than for positive (*k* = 2, *M* = 19.98, *SD* = 7.49) images (*t*(102) = 6.47, *p* < .01).

### Results

To account for the nested design of our data (i.e., 40 ratings of affect nested within each individual, including two images per valence and ten repetitions per image), we predicted affect from valence (2: positive vs. negative) and repetition (continuous: 1–10) using Multilevel Modeling (MLM) in R (version 4.0.3; Pinheiro et al., 2020; R Core Team, 2020). Preliminary analyses indicated that a two-level model with fixed effects for valence and repetition, and random effects for intercepts (e.g., allowing different affect baselines per participant) and slopes (e.g., allowing different associations between any two variables per participant) best fitted our data and constituted our base model.

#### Testing the Differential Habituation Hypothesis

To test whether repeated stimulus exposure leads to a faster decline in affect for positive (vs. negative) images, we predicted affect from image valence (2: positive vs. negative; fixed), repetition (continuous, 1–10; fixed), Valence×Repetition (fixed), with random intercepts and slopes. In omnibus tests, valence was effects-coded (negative = −1, positive = 1) and repetition (1–10) was person-centered on time 1, to observe how affect elicited by the very first image presentation changed with repeated presentations of the same image for each individual. Because gender was not related to anxiety symptomatology in this sample (β = .03, *p* = .86) and we did not have a priori hypothesis about gender differences in habituation, therefore, analyses including gender will not be discussed further.^3^

The MLM results showed a main effect of image valence, *t*(4,041) = 7.04, β = −5.07, *p* < .001, indicating that negative images elicited stronger affective reactions on average than positive images. Second, the main effect of repetition showed the general habituation effect: emotional reactions lost their strength with repeated exposure, *t*(4,041) = 5.84, β = −.45, *p* < .001.

**Figure 2.**
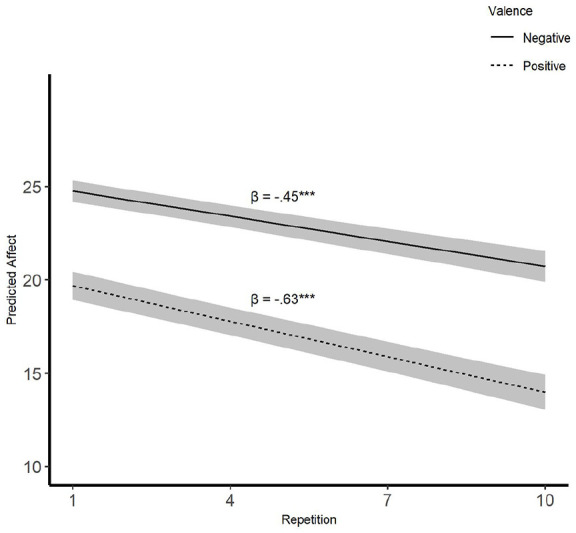
Habituation trajectories as a function of stimuli valence. *Note.* Rate of habituation to positive and negative stimuli. The bands around predicted lines represent 1SE above and below the regression line. ***p < .001.

Most importantly, we found support for the differential-habituation hypothesis, as the Repetition×Valence interaction was significant, *t*(4,041) = 2.22, β = −.19, *p* = .03. As predicted, habituation occurred significantly faster for positive, *t*(4,041) = 7.43, β = −.63, *p* < .001, than for negative, *t*(4,041) = 5.84, β = −.45, *p* < .001, images (see Figure 2). This interaction remained significant controlling for baseline affect (i.e., affect after the first repetition of each image), *t*(16,665) = 2.21, β = −.14, *p* = .03, which indicated that valence differences in habituation rates cannot be explained by baseline differences in affect (i.e., the initial affective reaction being stronger to negative images than positive; see Supplementary Materials for further analyses with baseline affect).

#### Testing the Anxiety Potentiation Hypothesis

Next, we tested the hypothesis that differential habituation gets further potentiated with anxiety by running our base model with the addition of a fixed effect term for anxiety symptomatology (standardized) and all higher-level interaction terms among valence, repetition, and anxiety symptomatology. Because our hypothesis was specific to the Valence×Repetition× Anxiety interaction, we focus our discussion below on the three-way interaction term; main effects and all lower-level interaction terms from this analysis are presented in Table 2.

**Table 2.** Parameter Estimates and Inferential Statistics from Analyses Testing Anxiety Symptomatology as a Moderator.

Predictors	Estimates		Inferential statistics		
	β	*SE*	*df*	*t*-value	*p*-value
Valence (effect coded)	−5.07	0.72	4,038	7.01	<.001
Repetition (first rep. centered)	−0.45	0.08	4,038	5.83	<.001
Anxiety	−0.07	0.65	102	0.11	.91
Valence×Repetition	−0.19	0.08	4,038	2.28	.02
Valence×Anxiety	0.24	0.82	4,038	0.29	.77
Repetition×Anxiety	0.05	0.09	4,038	0.53	.60
Valence×Repetition×Anxiety	−0.23	0.09	4,038	2.53	.01

**Figure 3.**
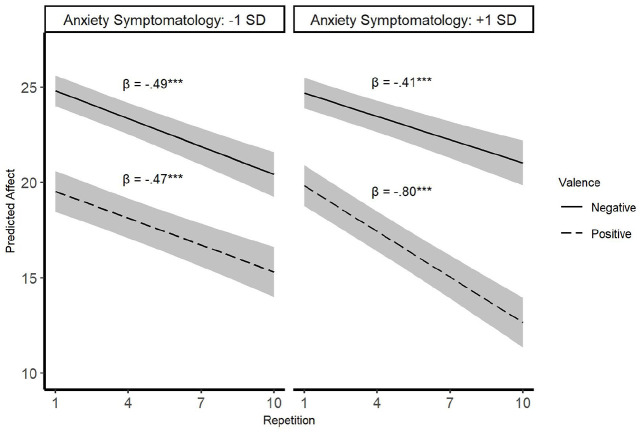
Habituation trajectories as a function of stimuli valence and anxiety. *Note*. Rate of habituation to positive and negative stimuli for individuals low (−1 SD below) and high (+1 SD above) the mean on anxiety symptomatology. The bands around predicted lines represent 1SE above and below the regression line. The *y*-axis (0–30) is truncated in the figure for illustrative purposes. ***p < .001.

This analysis yielded a significant three-way interaction between valence, repetition, and anxiety symptomatology, *t*(4,038) = 2.53, β = −.23, *p* = .01. To decompose this three-way interaction, we examined the two-way interaction between valence and repetition for people low (1 SD below the mean) versus high (1 SD above the mean) in anxiety symptomatology (see Figure 3).

Among people with *low* anxiety symptoms (1 SD below the mean), the results showed significant main effects for valence (i.e., stronger reactions to negative than positive; *t*(4,038) = 5.18, β = −5.28, *p* < .001) and repetition (i.e., lower affect over time; *t*(4,038) = 4.51, β = −.49, *p* < .001). However, the Valence×Repetition interaction was not significant, *t*(4,038) = 0.17, β = .02, *p* = .87, indicating similar habituation rates to positive versus negative stimuli.

In contrast, among people reporting *high* levels of anxiety (1 SD above the mean), not only the main effects of valence (*t*(4,038) = 4.76, β = −4.86, *p* < .001) and repetition (*t*(4,038) = 3.76, β = −.41, *p* < .001) but also the Valence×Repetition interaction were significant, *t*(4,038) = 3.40, β = −.39, *p* < .001. This interaction shows that individuals high in anxiety habituated faster to positive (*t*(4,038) = 6.72, β = −.80, *p* < .001) than to negative (*t*(4,038) = 3.76, β = −.41, *p* < .001) stimuli.

To further unpack the three-way interaction among valence, repetition, and anxiety symptomatology, we also ran simple slope analyses where we probed the Repetition× Anxiety interaction separately for negative vs. positive stimuli. This interaction was not significant for negative images (*t*(1,964) = 0.55, β = .05, *p* = .58), indicating that people high versus low in anxiety habituated at similar rates to negative stimuli. In contrast, the Repetition×Anxiety interaction was significant for positive stimuli, *t*(1,972) = 1.96, β = −.19, *p* = .05, demonstrating that highly anxious individuals habituated significantly faster than individuals with low anxiety levels (βs = −.80 and −.47, respectively). In other words, anxiety potentiation occurred primarily due to differences in positive habituation whereas our original hypothesis was that it would occur for both positive and negative habituation.

## Study 2: Pre-registered Replication with Normatively Selected Stimuli

Results from Study 1 provided full support for the differential habituation hypothesis and partial for the anxiety potentiation hypothesis. The overarching goal of Study 2 was to replicate and extend the findings from Study 1. First and most important, Study 2 aimed to replicate the differential habituation hypothesis. Second, we were interested in a deeper understanding of the role of anxiety in habituation. In Study 1, anxiety symptomatology was measured as an individual difference variable. Therefore, it was unclear whether anxiety might have a causal effect on habituation, such that higher anxiety levels affect individual’s emotional processing of positive (compared to negative) information. Therefore, in Study 2, we manipulated state anxiety right before the habituation trials to further examine how anxiety modulates habituation trajectories to positive and negative emotional elicitors. Furthermore, in Study 1, anxiety moderated habituation to only positive images, and we wanted to replicate this unexpected finding.

Third, we adopted a different methodological approach in Study 2 to gain better control over the stimuli used in the habituation task. Unlike Study 1 where participants were exposed to their own personalized set of images based on their highest affect ratings from the pilot phase, Study 2 utilized two pre-selected sets of stimuli shown to all participants. While the personalized approach in Study 1 allowed for tailored emotion elicitation, it limited the generalizability of findings beyond idiographic stimuli. Therefore, in Study 2, we standardized the stimuli by using the same set of 4 negative and 4 positive images across all participants. Negative images were selected to depict themes related to fear (2) or sadness (2) and positive images to love (2) or excitement (2) (see methods and Supplementary Materials for details on emotion themes).

We pre-registered the differential habituation hypothesis as confirmatory and the null effect of thematic differences within negative (sadness vs. fear) and positive (love vs. excitement) as exploratory (reported in the Supplementary Materials). The anxiety potentiation hypothesis was pre-registered in the following two ways: First, we expected experimental condition (2: anxiety vs. control) to moderate differential habituation such that positive-negative habituation differences would be more pronounced in the anxiety than in the control condition. Second, since the control condition in Study 2 was conceptually the closest to the design of Study 1, we pre-registered the hypothesis that positive-negative habituation differences would be potentiated by individual differences in anxiety symptomatology (measured in the same way as in Study 1) in the control condition.

## Methods

This study’s hypotheses, design, and analyses were pre-registered at AsPredicted.org (#68,077), and this initial pre-registration was revised before data collection started (#73,120). We also pre-registered an amendment to our exclusion criteria and sample size during data collection but before data analysis (#81,499).

### Participants

To achieve approximately 80% power, we sought to recruit a larger sample than Study 1, aiming for 100 participants per experimental condition (anxiety vs. control) with an even split between men and women, thus 50 participants per condition for each gender (see Simmons et al., 2013; VanVoorhis & Morgan, 2007). Considering the potential for higher attrition for a 2-session online study, we oversampled and recruited 268 participants from the same paid subject pool with the same eligibility criteria as Study 1.

Based on our pre-registered exclusion criteria (#73,120, #81,499), we excluded 14 participants because they failed to meet the attention check criteria (error rates exceeding 66% in embedded attention check items within session 1, or error rates exceeding 25% in a stimuli recognition task after the habituation trials within session 2). Additionally, 15 participants were excluded from the analyses due to incomplete completion of the habituation task (e.g., lack of cursor movement for the majority of the task duration).^4^

Following exclusions, the final sample included 239 participants (experimental group: 64 men and 67 women; control group: 54 men and 54 women). The average age of the sample was 21.02 years old (Med = 20, *SD* = 3.18), with the following race and ethnicity characteristics: 57.74% Asian or Pacific Islander, 26.36% White or Caucasian, 1.26% African-American, 7.54% mixed race and 6.69% other.

### Procedure

Data were collected in two online sessions completed 1 to 4 days apart. The first session was administered to all study participants on the Qualtrics survey platform and consisted of signing a consent form, filling out a demographic questionnaire, and answering a set of questionnaires, including the same three scales we had used in Study 1 to measure individual differences in anxiety symptomatology.

The following morning, participants received an email invitation to complete the second session. For increased believability of the experimental manipulation of stress, the second session was administered during working days and hours. At the onset of the second session, participants were randomly assigned to either the experimental or the control condition (separately within each gender) and were sent to the *Inquisit by Millisecond* experiment platform where they completed the stress manipulation and the habituation task.

The *Inquisit* session started with questions measuring state emotions (anxiety, excitement, sadness, calmness, and interest) to obtain a pre-manipulation rating of baseline anxiety. Next participants completed a practice rating task, comprising 4 negative, 4 positive, and 4 neutral images. This practice task paralleled the Phase 1 of Study 1 by allowing participants to calibrate their use of the rating scale before they began the habituation task. As in Study 1, neutral images were only included in the practice task.

Next, participants completed the “My Dream Job” task intended to manipulate state anxiety. In the experimental (high state anxiety) group, participants were told to imagine that they got an interview for their dream job and were asked to deliver a speech about their strengths and weaknesses for the position to a panel of two public-speaking experts trained in speech evaluation via a live Zoom meeting. This step was necessary to create a moderately stressful situation and was adapted from the Trier Social Stress Test (Kirschbaum et al., 1993), which is designed to elicit public-speaking anxiety. Participants were given 1.5 min to mentally prepare for delivering a 5 min speech describing why they would be a good candidate for their ideal job.

We included an “active” control condition where participants were also asked to imagine that they got an interview for their dream job but that they would simply write an essay about the qualities that would make them well-suited for this job and that their essay would be reviewed by a computer algorithm for content and style (modified from Birkett, 2011; Kirschbaum et al., 1993). To make the experience as parallel to the experimental group as possible, control participants were also given 1.5 min to mentally prepare for writing the essay. Both groups were told that the Dream Job Task was going to take place as the last task of the session, following the image rating task.

Immediately after the preparation period, participants in both groups rated their state anxiety (i.e., post-manipulation anxiety) on the same item used to measure baseline anxiety. These two measures allowed us to conduct a within-subject manipulation check on state anxiety levels in both the experimental and the control group.

**Figure 4.**
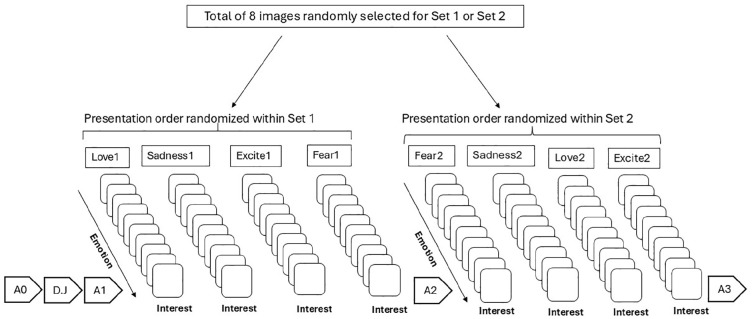
Flowchart of experimental design in Study 2. *Note*. Study 2 flowchart of the second study session, in which state anxiety (A) was measured at baseline (A0) and at several timepoints throughout the habituation paradigm (A1, A2, A3). D.J represents the My Dream Job task.

Subsequently, participants completed the habituation task where they rated their affective reactions to the eight images selected from the IAPS (Lang et al., 2008; see the Habituation Stimuli section for details). Images were randomized into two sets such that each set included one image from each theme category (i.e., love, excitement, fear, and sadness). Images within a set were presented in randomized blocks with 10 repetitions of 5 s per image. Participants were instructed to look at each image for the full time it was presented and to rate affective reaction to the image after each presentation (see Figure 4 for study design).

Additionally, after the tenth repetition of each image, participants were asked to rate their current level of interest in and engagement with the task. We used interest ratings to rule out the possibility that habituation effects were simply due to disengagement from the task. Lastly, participants were sent back to Qualtrics where they completed a short attention check task, were probed for suspicions about the goals of the study, and then debriefed about the deception since no one gave the 5-minute speech or wrote the essay. No participants were excluded because of suspicions about the nature of the experiment.

#### Habituation Stimuli Selection

As in Study 1, stimuli were selected from the IAPS set based on pre-established norms (Ito et al., 1998; valence: 1 = *maximum negativity*, 9 = *maximum positivity*). Negative (*k* = 4, *M* = 7.32, *SD* = 1.83) and positive (*k* = 4, *M* = 2.05, *SD* = 1.51) image sets were matched on arousal (*M*_neg_ = 5.98, *SD*_neg_ = 0.66; *M*_pos_ = 6.65, *SD*_pos_ = 0.99, *t*(5.24) = 1.13, *p* = .31).

### Measures

Anxiety symptomatology was measured in Session 1, prior to the habituation trials in Session 2; all other measures were administered in Session 2.

#### Anxiety Symptomatology

Individual differences in anxiety were measured using the same scales as in Study 1 (see pre-registration): BAI (α = .95, *M* = 19.88, *SD* = 16.41); DASS-21 anxiety (α = .82, *M* = 6.34, *SD* = 7.03) and DASS-21 stress (α = .87, *M* = 10.79; *SD* = 8.95). The three scales were highly correlated (all *r*s > .76) and were thus standardized and averaged into a single anxiety composite (α *=* 0.92).

#### Affect During Habituation

We measured affect during habituation in the same way as in Study 1. Participants were given 5 s to look at each presented image and then rated the valence and intensity of their affective reaction to the image (“How does this image make you feel right now, at this moment?”) on a sliding scale from −3 (*extremely negative*) to 3 (*extremely positive*) with increments of 0.1, which were transformed into numerical values ranging from −30 to +30. As described in Study 1, affect ratings to negative stimuli were multiplied by −1 so that higher numbers indicate both higher negative and higher positive affect. Identical to Study 1, participants’ affective responses to the first presentation of each image during the habituation task were used to operationalize baseline affect (*M* = 14.32; *SD* = 12.19; range: −30–30). As in Study 1, baseline affect was stronger (*t*(238) = 12.73, *p* < .01) for negative (*M* = 18.42, *SD* = 7.77) than positive (*M* = 10.21; *SD* = 8.46) images.

#### State Emotions Throughout Session 2

##### Anxiety

State anxiety was measured by asking “Right now, how tense, on edge, or anxious do you feel?” Participants chose a rating on a sliding scale from 1 (*not at all*), 4 (*moderately so*), to 7 (*very much so*) with increments of 0.1. This question was asked 4 times throughout Session 2, as illustrated in Figure 4 above): (a) at the very beginning of Session 2, that is, *baseline anxiety* (*M* = 2.57, *SD* = 1.46); (b) right after the “My Dream Job” task and immediately before the habituation task, that is, *post-manipulation anxiety* (*M* = 4.14, *SD* = 1.83); (c) mid-way during the habituation task, after the first set of images were rated, that is, *mid-habituation anxiety* (*M* = 3.71, *SD* = 1.84); and (d) right after the habituation task, that is, *post-habituation anxiety* (*M* = 3.49, *SD* = 1.99; see Manipulation Checks below).

##### Interest

To rule out the possibility that reduction in emotional response is due to loss of interest in the task, we measured level of interest and used it as a covariate in key analyses. Using a sliding scale from 1 (*not at all*), 4 (*moderately so*), to 7 (*very much so*) with increments of 0.1, participants responded to the question “How interested, engaged, & attentive do you feel at this moment?” at the very beginning of Session 2 (Baseline interest: *M* = 4.09, *SD* = 1.31) and 8 times throughout the habituation task, always following the 10th presentation of each image. The latter were averaged into a single composite to index habituation interest (*M* = 2.49, *SD* = 1.08, *α* = 95; See the Supplementary Materials for additional analyses on interest).

## Results

### Manipulation Check on State Anxiety

**Figure 5.**
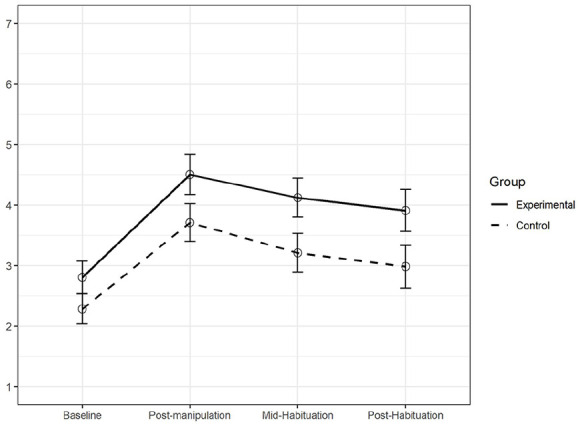
Trajectory of state anxiety in Study 2. *Note.* Bars represent 95% CIs.

Figure 5 shows the mean levels of state anxiety at four times throughout Session 2, separately for the participants in the experimental and the control group. The critical comparison for our purposes was between anxiety at baseline (at the very beginning of the experimental session) and right after the manipulation.

Anxiety was higher in the experimental than in the control group at baseline (*M*_E_ = 2.80, *SD*_E_ = 1.55; *M*_C_ = 2.28, *SD*_C_ = 1.29; *t*(236) = 2.83, *p* < .01) and at post manipulation (*M*_E_ = 4.5, *SD*_E_ = 1.91; *M*_C_ = 3.71, *SD*_C_ = 1.64; *t*(236.63) = 3.44, *p* < .001). Additionally, anxiety levels increased from baseline to post-manipulation significantly in both groups (*t*_E_(249.13) = 7.88, *p* < .001; *t*_C_(202.88) = 7.11, *p* < .001) with no significant group differences in this increase (*t*_diff_(235.83) = 1.06, *p* = .29).

Baseline differences in anxiety between the experimental conditions were unexpected. However, since the study was entirely computer-delivered and procedures were identical up until the dream job task (and randomization occurred at the very beginning of Session 2), we assume these differences were not due to any systematic bias (e.g., experimenter demand). Another unexpected finding concerned the significant increase in anxiety reported in the control group. When we examined written responses to open-ended questions asked during funnel debriefing (e.g., What do you believe is the purpose of this study? Did you find anything strange in the study procedure?), we observed that some participants in the control group had indeed spontaneously indicated they had been quite stressed. For example, one wrote “I also found it interesting how the images calmed me down for the supposed dream job task, which at first seemed daunting, especially since it was meant to be graded by a computer algorithm.”

Because both groups experienced a significant increase in anxiety due to the “My Dream Job Task” and because post-manipulation anxiety is the psychological mechanism the experimental condition was trying to manipulate in the first place, we followed our pre-registered analyses plan (#73,120) and used individual differences in post-manipulation anxiety ratings (instead of experimental condition), as the key moderator in testing the anxiety potentiation hypothesis. In additional analyses, we also controlled for baseline anxiety to rule out that any effect of post-manipulation anxiety on habituation is due to baseline differences.

### Confirmatory Analyses

Similar to Study 1, we performed MLM to account for the nested design of the study. Specifically, for each individual, 10 repetitions were nested within 8 images, nested within themes (4: love, excitement, fear, sadness), nested within valence (2: Positive vs. Negative). Preliminary analyses indicated that the base model used in Study 1 fit Study 2 data best as well (i.e., fixed effects for valence and repetition, and random effects for intercepts and slopes).

#### Replicating the Differential Habituation Pattern from Study 1

Affect was predicted from valence (2: positive vs. negative; fixed), repetition (continuous, 1–10; fixed), Valence× Repetition (fixed), with random intercepts and slopes. Once again, valence was effects-coded (negative = −1, positive = 1) and repetition (1–10) was person-centered on time 1, to observe changes in affect with repeated exposure to images within individual.

Closely replicating Study 1, positive images elicited weaker affective reactions on average than negative images (valence: *t*(18,878) = 14.25, β = −8.79, *p* < .001), and the magnitude of affective reactions (across image valence) got smaller with repeated exposure (repetition: *t*(18,878) = 5.11, β = −.25, *p* < .001). Supporting the differential habituation hypothesis, the two-way Repetition×Valence interaction was also replicated, *t*(18,878) = 1.99, β = −.12, *p* = .047, with habituation occurring significantly faster for positive images (*t*(18,878) = 7.19, β = −.37, *p* < .001) than for negative images (*t*(18,878) = 5.11, β = −.25, *p* < .001).

In additional analyses, we aimed to rule out boredom and thus, disengagement from the task as an alternative explanation for the reduction in the strength of participants’ affective reactions across trials. Therefore, we ran the above-described model while controlling for interest ratings. The two-way Repetition×Valence interaction remained significant (*t*(18,397) = 2.09, β = −.12, *p* = .04).

#### Testing the Anxiety Potentiation Hypothesis with Situationally Induced Anxiety

As would be expected from the failed manipulation check analyses, when we used experimental condition (2: anxiety vs. control) in testing the anxiety potentiation hypothesis, the 3-way interaction between Valence× Repetition×Condition was not significant (*t*(18,875) = .44, *b* = −.05, *p* = .66). Therefore, in a second iteration, we replaced experimental condition with post-manipulation state anxiety scores (pre-registered in #73,120).

**Figure 6.**
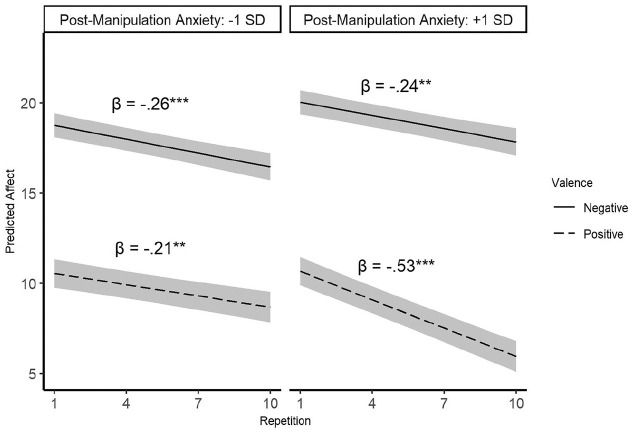
Habituation trajectories as a function of stimuli valence and post-manipulation anxiety. *Note.* Rate of habituation to positive and negative stimuli for individuals low (−1 SD below) and high (+1 SD above) the mean on post-manipulation anxiety. The bands around predicted lines represent 1SE above and below the regression line. **p < .01. ***p < .001.

As presented in Figure 6, results indicated a significant three-way interaction between valence, repetition, and anxiety, *t*(18,875) = 2.87, β = −.09, *p* = .004 which remained virtually unchanged when controlling for baseline anxiety, *t*(18,796) = 2.88, *b* = −-0.09, *p* = .004. Further analyses showed that the two-way interaction between valence and repetition was significant only for individuals high (1 SD above the mean) in anxiety, *t*(18,875) = 3.46, *b* = −0.28, *p* < .001, with faster habituation to positive (*t*(18,875) = 7.43, *b* = −0.53, *p* < .001) than negative (*t*(18,875) = 3.51, *b* = −0.24, *p* < .001) images. Among individuals low in post-manipulation anxiety (1 SD below the mean), results indicated significant effects for valence (i.e., higher intensity for negative vs. positive images; *t*(18,875) = 9.41, *b* = −8.21, *p* < .001) and for repetition (i.e., decreased valence intensity over time; *t*(18,875) = 3.69, *b* = −0.26, *p* < .001). However, the 2-way interaction between Valence×Repetition was not significant, *t*(18,875) = 0.60, *b* = 0.05, *p* = .55, indicating similar trajectories for negative and positive habituation.

In additional simple slope analyses, we examined the two-way interaction of Repetition×Post Manipulation Anxiety separately for negative and positive stimuli. Repetition× Anxiety interaction was not significant for negative images, *t*(18,875) = 0.13, β = .003, *p* = .90, indicating that participants habituated to negative stimuli similarly regardless of anxiety after the manipulation. In contrast, the 2-way interaction between repetition and anxiety was significant for positively valenced stimuli, *t*(18,875) = 3.18, β = −.09, *p* = .004, demonstrating that individuals with high (+1 SD) anxiety levels habituated (*t*(18,875) = 7.43, β = −.52, *p* < .001) significantly faster than individuals with low (−1 SD) anxiety levels (*t*(18,875) = 2.93, β = −.21, *p* < .001). This pattern of results was identical to findings from Study 1.

#### Replicating Study 1 Findings on Potentiation of Valence Differences by Anxiety Symptomatology

To examine whether the significant Repetition×Valence×Anxiety symptomatology interaction from Study 1 could be conceptually replicated, we tested the three-way interaction between repetition (continuous, 1–-10; fixed), valence (2: positive vs. negative; fixed), and anxiety symptomatology measured the day before the experiment (using the same measure as Study 1) on affect ratings. We constrained this analysis to the participants in the control group because the design of the control group is theoretically more analogous to the design of Study 1. This analytic approach had been pre-registered in our analysis plan (#73,120).

We ran our base model of valence and repetition, including anxious symptomatology, all higher-level interaction terms, and random effects for effect-coded valence and repetition. Replicating Study 1, the results revealed a marginal three-way interaction, *t*(8,526) = 1.87, *b* = −0.18, *p* = .06. Although this effect did not reach conventional statistical significance (*p* < .05), it was consistent with our pre-registered theoretical expectations; therefore, we proceeded with breaking down the interaction.

Among those higher in anxious symptomatology, there was a significant Valence×Repetition interaction, *t*(8,526) = 2.14, *b* = −0.25, *p* = .03, indicating significantly faster habituation to positive (*t*(8,526) = 5.20, β = −.54, *p* < .001) than to negative (*t*(8,526) = 2.46, β = −.30, *p* = .01) stimuli. Similar to Study 1, the two-way interaction was not significant for individuals with lower levels of anxious symptomatology, *t*(8,526) = 0.42, β = .04, *p* = .68, indicating comparable habituation rates to positive versus negative stimuli.

## General Discussion

The present research examined two key hypotheses about affective habituation that emotional reactions should decrease at a slower rate for negative than positive stimuli (differential habituation hypothesis) and that anxiety should further potentiate this normative difference (anxiety potentiation hypothesis). Results from two experiments were largely consistent with these expectations.

The rationale for the differential habituation hypothesis was drawn from a large and diverse body of research showing that humans prioritize processing of cues related to threat over rewards because of their differential value for survival (Öhman, 2009; Rozin & Royzman, 2001). That positive cues are less relevant to survival suggests that when such cues are presented repeatedly, their informational value (for survival) should decay more rapidly, leading them to lose their capacity to elicit emotional reactions much faster than negative cues. Indeed, our findings showed that although the intensity of people’s affective reactions to both positive and negative stimuli declined linearly with repeated exposure, this decline was significantly faster for positive (vs. negative) stimuli (Studies 1 and 2).

Importantly, this normative difference in positive vs. negative habituation rates was further enhanced by anxiety both when it was measured as a pre-existing individual difference variable (Studies 1 and 2) and when it was situationally induced (Study 2), supporting the anxiety potentiation hypothesis. However, the exact pattern in which this potentiation occurred was only partially consistent with our initial hypotheses. Since anxiety entails a heightened sense of threat (Beck & Clark, 1997), and because under threat positive information may be deemed even less pertinent to survival whereas negative information becomes all the more important, our initial expectation was for anxiety to moderate both positive and negative habituation. However, Study 1 results showed significant moderation only for positive images—a finding that was pre-registered and replicated in Study 2.

Why did anxiety not moderate habituation to threat information given everything we know about negative information processing biases associated with anxiety (Mathews & MacLeod, 2002)? Several explanations come to mind. First, anxiety might impact attention and cognition at earlier phases of information processing (e.g., vigilance, orienting) than habituation so that biases related to avoiding or persevering on threat cues typically associated with anxiety are bypassed. Another potential explanation is that anxiety affects only positive habituation because negative information, due to its normative relevance to survival, creates a situation strong enough to override individual differences in anxiety (e.g., de Jong et al., 2010). It is also possible that the tendency of individuals higher in anxiety to expect negative outcomes (e.g., Cabeleira et al., 2014) leads to a compensatory dampening of negative affect during repeated exposure to negative stimuli, thus, minimizing group differences in negative habituation. Finally, the differences could also partly be explained by the fact that we operationalized anxiety in terms of short-term anxiety symptoms (experienced in the last 2 weeks or situationally induced) in a non-selected sample, rather than in a clinical sample. It will be important for future research to examine how anxiety modulates habituation using different paradigms and affect measures and to explore whether the specificity of anxiety’s effect on positive habituation generalizes in other contexts or populations.

### Implications

Among the key implications of these findings for research and theory on emotion is that the cognitive mechanisms humans have developed to be able to rapidly respond to threats come at the cost of not being able to savor rewarding experiences. In other words, the drive toward achieving hedonic balance (more positive and less negative emotions) that is central to people’s sense of well-being (e.g., Diener et al., 1999) runs an uphill battle against the evolutionary imperative to pay more attention to threats to escape injury and death. However, our findings on anxiety potentiation also show that there are important individual differences in this general evolutionary pattern to habituate faster to reward (vs. threat) information, which might result in some individuals being better able to balance the need to survive and the desire to enjoy life.

The current findings also have important implications for our understanding of emotional dysfunction in clinical disorders. The potential role of positive emotions in vulnerability to mood and anxiety disorders is a topic relatively neglected compared to the emphasis placed on the role of negative emotions in psychopathology (see Gross & Jazaieri, 2014 for review). For example, heightened negative affect is emphasized as the central feature in a wide range of psychological disorders (Kring & Bachorowski, 1999; Nolen-Hoeksema, 2000). Likewise, common psychotherapy methods, such as cognitive components of the CBT, emphasize negatively biased cognition as a core element in the treatment of mood and anxiety disorders, concentrating on patients’ daily negative affect and related automatic thoughts (Young et al., 2014).

In contrast, although experiencing prolonged positive emotion is known to enhance physical, social, and intellectual resources (e.g., Fredrickson & Losada, 2005), positive habituation, alongside positive information processing biases more generally, has received relatively little attention in research on psychopathology. Our findings underscore the importance of addressing this gap in future research. For example, it will be important to better understand the causal dynamics between anxiety and positive habituation, particularly the question of whether they reciprocally maintain and strengthen each other. Knowing whether faster positive habituation feeds back into anxiety would allow researchers to develop interventions that can target this process specifically.

These findings also have implications for research on hedonic adaptation. By its very nature, the question of how people adapt to extreme life events over the course of months or years necessitates observational data where positive and negative events almost always occur at the between-subjects level (e.g., it is very unlikely for the same person to win the lottery *and* survive a plane crash). Existing data for hedonic adaptation theories’ long-standing argument that “bad are stronger than good” (e.g., Baumeister et al., 2001) therefore, remains inconclusive. Although affective habituation and hedonic adaptation examine processes happening at very different time scales, they both examine the duration of affective states. As such, the current findings provide experimental evidence that is consistent with the argument that people adapt to positive life events much faster than they adapt to negative life events.

Despite subtle conceptual differences between the valence of a stimulus and its subjective enjoyment (e.g., a comedy can elicit boredom), our findings on positive habituation align with and extend the hedonic decline literature (Epstein et al., 2009; Higgs et al., 2008; as cited in Galak & Redden, 2018). This convergence is not entirely unexpected, given that sensory-specific satiety is often considered a specific instance of affective habituation (Epstein et al., 2009; Higgs et al., 2008, as cited in Galak & Redden, 2018). Nevertheless, our findings offer a theoretical contribution by demonstrating positive habituation beyond sensory-specific satiety and by enabling direct comparisons with negative habituation.

### Strengths and Limitations

Several strengths of the current research are noteworthy. First, even though each study’s design had its own strengths and limitations, when put together Studies 1 and 2 findings converged, strengthening our confidence in the evidentiary value of the results. Because in Study 1 experimental stimuli were idiographically normed (i.e., subjectively rated as the most pleasant vs. unpleasant for each individual), the psychological meaning of the stimuli was equated across participants, which helped account for individual differences in responses to stimuli and increased ecological validity of the findings. In contrast, Study 2 used a fixed set of images selected on the basis of pre-established normative ratings to ensure that the findings generalized beyond idiographic stimuli (though the effect sizes were smaller). Furthermore, both studies showed that the differential habituation hypothesis generalized across different types of stimuli (see Supplementary Materials), Study 1 by coding for different affective themes present in the images viewed by each participant, and Study 2 by experimentally varying affective themes within each valence category (love vs. excitement and fear vs. sadness). Despite this convergence, these effects should be interpreted with caution because experiments conducted under controlled conditions tend to overestimate effect sizes.

The question of how anxiety might modulate habituation was also triangulated by different methods in the two studies. Whereas in Study 1, anxiety symptomatology during the past week was simply measured, Study 2 experimentally induced state anxiety. Despite the strength inherent in the design of Study 2, however, individual differences in post-manipulation anxiety, rather than experimental condition per se, significantly moderated the Valence×Repetition interaction on affect. This is likely due to the manipulation unexpectedly increasing anxiety both in the control and experimental conditions. Therefore, although the pattern of findings with post-manipulation anxiety was consistent with our pre-registered hypotheses, and Study 1 findings were replicated in the control condition with pre-existing anxiety symptomatology, we need to be cautious in the causal inferences we make about the role of anxiety. As we discussed earlier, tackling the causality question should be a priority in future work because just as it is important to know if anxiety leads people to habituate faster to positive stimuli, it is also important to know if faster habituation to positive elicitors serves as a precursor to developing anxiety symptoms.

As with all research using self-report measures, affect ratings used as the main outcome might be subject to people misreporting their experiences. Although individual differences in reporting biases were controlled to some extent by multi-level modeling, the findings should nevertheless be replicated with additional objective measures of habituation (e.g., skin conductance) in future studies.

Finally, our samples were drawn from a population of predominantly Asian- and European-American youth attending college in the United States. As such, our studies do not address whether the same effects would hold in older, less educated groups, in other racial/ethnic groups in the United States, or in different cultures.

## Conclusions

Temporal dynamics of affective responding is a topic that is receiving growing empirical attention (see Kuppens, 2015 for a special issue), being examined in multiple and diverse frameworks, including that of hedonic adaptation, affective chronometry, and affective habituation. The current research contributes to this broader aim of understanding the time course of emotional responding by focusing on the moment-by-moment affective habituation to repeated emotion elicitation. Specifically, by showing that people habituate to positive stimuli faster than to negative stimuli, and the size of this differential valence effect increases with anxiety, the current results suggest that the way people respond to positive stimuli is of importance as it might serve as an additional marker for vulnerability to emotional dysfunction.

## Supplemental Material

sj-docx-1-psp-10.1177_01461672251348486 – Supplemental material for Letting Go of the Negative, Holding on to the Positive? Within-Person Trajectories of Affective Habituation to Negative and Positive Stimuli Supplemental material, sj-docx-1-psp-10.1177_01461672251348486 for Letting Go of the Negative, Holding on to the Positive? Within-Person Trajectories of Affective Habituation to Negative and Positive Stimuli by Elizabeth Yartsev, Oliver P. John and Özlem N. Ayduk in Personality and Social Psychology Bulletin

## Notes

1. IAPS image numbers: (a) Neutral: 2210, 9210, 2200. 9070, 2410, 2190; (b) Negative: 6212, 6831, 8230, 6312, 2800, 9050, 3530, 6550, 3550, 3220; (c) Positive: 5621, 8031, 4599, 8030, 5628, 8080, 5629, 8370, 4601, 4641.

2. Analyses with each of the three anxiety scales separately yielded identical pattern of significant results with the composite measure of anxiety and are presented in the Supplementary Materials.

3. The three-way interaction between valence, repetition, and gender was not significant in either Study 1 (β = .03, *p* = .85) or Study 2 (β = .04, *p* = .52); thus, gender was not a significant moderator of differential habituation. The four-way interaction between valence, repetition, anxiety, and gender interaction was significant in Study 1, (β = −.42, *p* = .02) but not in Study 2 (β = .02, *p* = .83). Given this lack of replication and the fact that detecting a four-way interaction requires high statistical power, we refrain from interpreting the significant gender interaction further.

4. While the pre-registration (#81,499) initially specified the removal of participants in the experimental group who did not exhibit increased state anxiety in response to the manipulation, we ultimately decided to retain them in the sample because it’s a more conservative approach.
