# Supplementary material for: Letting Go of the Negative, Holding on to the Positive? Within-Person Trajectories of Affective Habituation to Negative and Positive Stimuli
Source: Pers Soc Psychol Bull. 2025 Aug 3;52(9):2966–80. doi: 10.1177/01461672251348486 (PMC13392163; doi:10.1177/01461672251348486)
Supplement: sj-docx-1-psp-10.1177_01461672251348486 – Supplemental material for Letting Go of the Negative, Holding on to the Positive? Within-Person Trajectories of Affective Habituation to Negative and Positive Stimuli [file sj-docx-1-psp-10.1177_01461672251348486.docx]

**Letting Go of the Negative, Holding on to the Positive?**

**Within-Person Trajectories of Affective Habituation to Negative and Positive Stimuli**

**SUPPLEMENTARY ONLINE MATERIALS**

Elizabeth Yartsev

Oliver P. John

Özlem N. Ayduk

University of California, Berkeley

**STUDY 1**

Part I lists additional measures which were administered for exploratory purposes. Part II includes analyses on additional moderators (e.g., depression). Part III reports additional analyses conducted on the thematic ratings of the experimental stimuli.

**Part I - Additional Measures for Study 1**

**Individual Differences Questionnaires**

The following measures were included in the survey part of Study 1:

- Center for Epidemiological Studies - Depression (CES-D, Radloff, 1977)
- Depression, Anxiety, & Distress Scale – 21 (DASS 21, Lovibond & Lovibond 1995)
- Rumination Response Scale (RRS, Treynor et al., 2003)
- Big-Five Inventory (BFI-44, John, Donahue, & Kentle, 1991).
- Emotion Regulation Questionnaire (ERQ, Gross & John, 2003),
- Experiences in Close Relationships Scale – Short Form (ECR-short, Wei et al., 2007)
- Rejection Sensitivity Questionnaire (RSQ, Downey & Feldman, 1996)
- Behavioral Inhibition and Behavioral Activation Scales (BIS-BAS, Carver & White, 1994)

**Discrete Emotions Elicited During Habituation**

In Phase 3 of the experiment, we presented participants with the 4 images they were exposed to in the habituation task they had previously completed. Participants were asked to choose from a drop-down menu the most dominant emotion they felt when they saw each of the 4 images for the first and the last time. Emotion options included: fear, sadness, disgust, anger, awe, excitement, contentment, amusement, anxiety, empathy, horror, interest, joy, nostalgia, satisfaction, romance, and other (see Frequencies in Table S1.1).

**
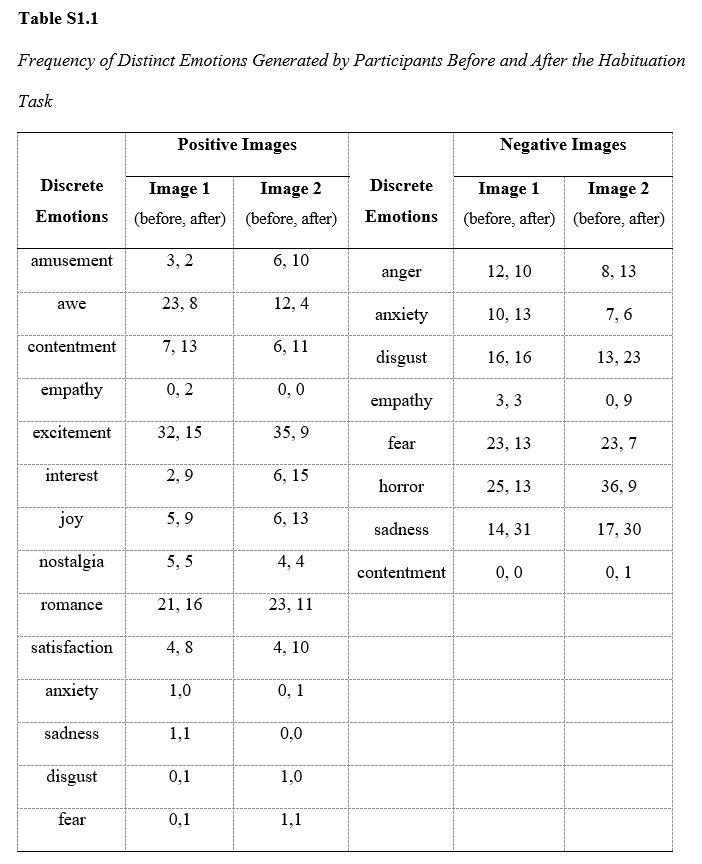
**

**Part II – Additional Moderator Analyses for Study 1**

**Moderation by Individual Scales of the Anxiety Symptomatology Composite**

Analyses with the anxiety composite reported in the manuscript were rerun with each of the individual anxiety scales that comprised the composite. Specifically, in 3 separate models, we predicted affect from standardized anxiety scores using the DASS 21 anxiety, DASS 21 stress and BAI, with repetition (continuous, 1-10; fixed) and valence (2: positive vs. negative; fixed).

**
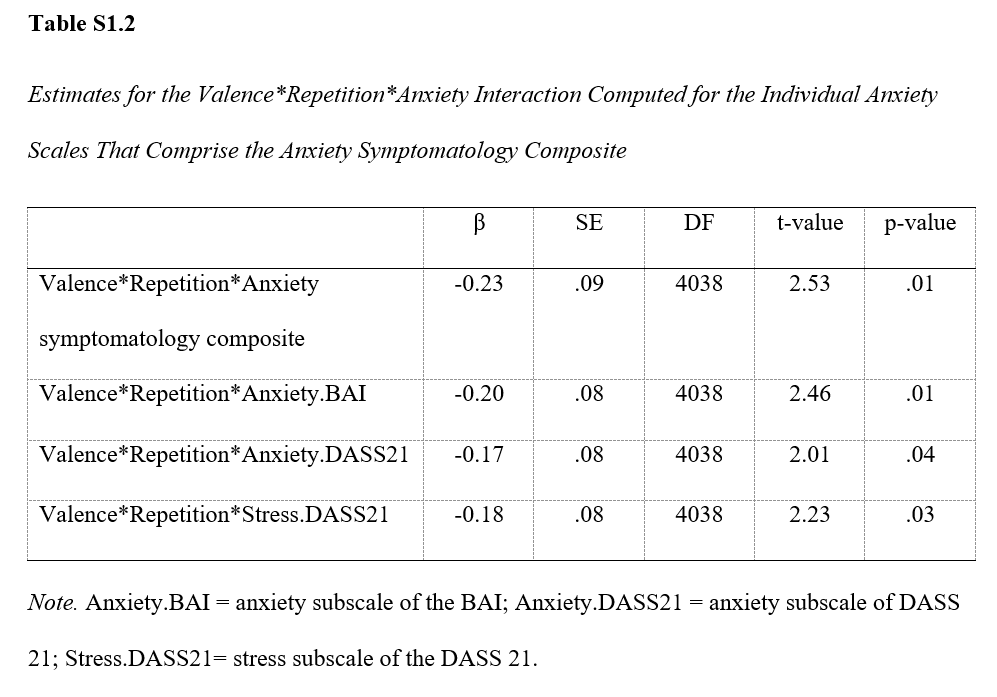
**

Results presented in Table S1.2 show that the anxiety*repetition*valence interaction which was our theoretical focus was statistically significant in all 3 cases. These results suggest that the 3-way interaction reported in the main manuscript was not primarily driven by any one scale that made up the composite and anxiety moderated habituation regardless of the scale used to operationalize it.

**Moderation by Depression**

We explored whether emotional habituation rates differ as a function of depression symptoms (measured by the CES-D). We repeated the key multilevel model of predicting affect from repetition (continuous, 1-10; fixed), valence (2: positive vs. negative; fixed) and depression (standardized, fixed) including all higher interaction terms among valence, repetition and depression, with random intercepts and slopes. Main effects and all lower-level interaction terms from this analysis are presented in Table S1.3 which shows that the pattern of findings for depression was similar to those reported in the main text for anxiety.


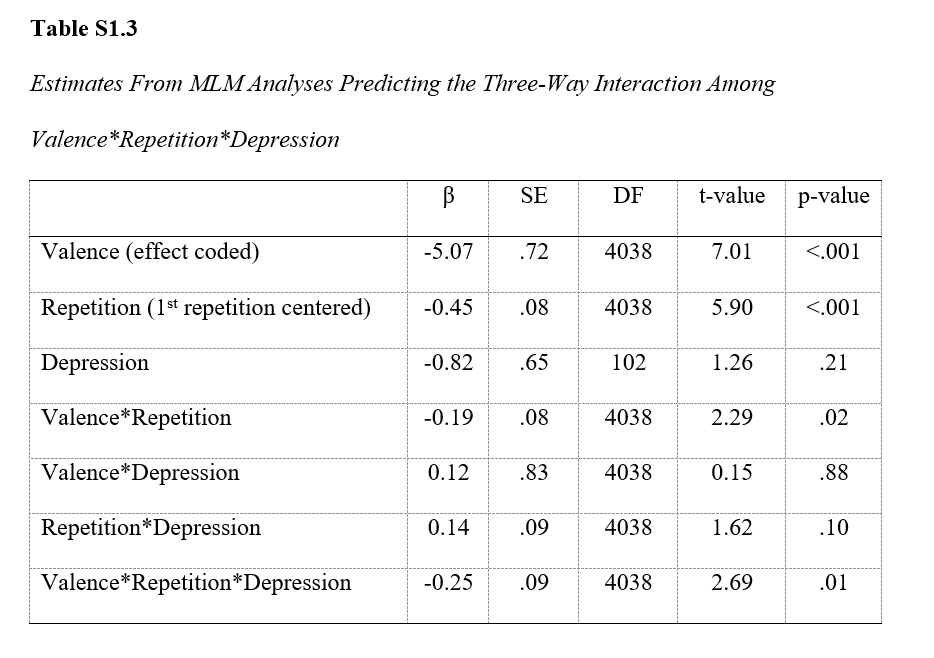


**Effect of Anxiety and Depression while Controlling for Each Other**

When the anxiety potentiation hypothesis was tested while controlling for depression scores, the 3-way interaction between valence, repetition and anxiety reported in the main text remained significant, *t*(4038)= 2.53, *b* = -0.23, *p* = 0.01. Similarly, the valence*repetition* depression interaction reported above remained significant when controlling for anxiety, *t*(4038)= 2.69, *β* = -0.25, *p* = .007.

**Moderation by Baseline Affect Intensity**

One question we wished to address was whether baseline affect intensity moderated subsequent rates of habituation. It is possible that stimuli that elicit stronger reactions to start with have more informational value for danger (negative) vs. safety (positive). When danger signals and safety signals are particularly strong, habituation to negative stimuli might slow down even further while positive habituation might further accelerate. Thus, the differential habituation pattern we hypothesized at the normative level might be even more pronounced for highly negative and positive stimuli. When stimuli elicit milder reactions and thus signal relatively low relevance either for safety or danger, valence differences in habituation rates might get smaller, leading to a weaker habituation pattern overall. Thus, the differential habituation pattern might not be present for stimuli that are mildly or weakly positive or negative.

To explore these possibilities, baseline affect was grand mean centered and was added as a fixed effect to our base model of valence and repetition predicting emotional responses. A single outlier as identified by R was excluded from models of baseline affect. This analysis yielded a significant 3-way interaction, *t*(4000) = 5.51, *β* = -.05, *p* < .001, indicating that people show different habituation rates for positive vs. negative images depending on baseline affect (see Figure S1.1). Note that the repetition*valence interaction remained significant, *t*(4000) = 4.00, *β* = -0.39, *p* < .001, even in the presence of this 3-way interaction.


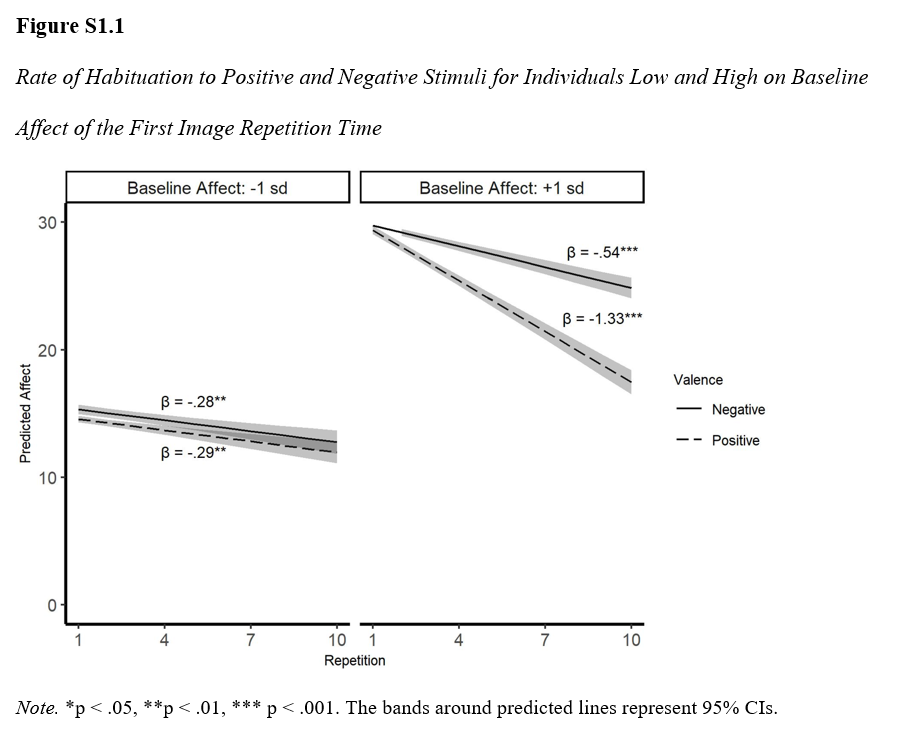


In subsequent simple slope analyses conducted to unpack this interaction, we examined valence*repetition interaction for images high (+1 SD) vs. low (-1 SD) in baseline affect intensity. For high intensity (+1 SD) images, there was significant 2-way interaction between valence*repetition (*t*(4000) = 6.55, *β* = -.78, *p* < .001) such that rate of habituation, although significant for both positive (*t*(4000)= 12.42, *β* = -1.33, *p* < .001) and negative (*t*(4000) = 6.32, *β* = -0.54, *p* < .001) stimuli, was much faster for the former than for the latter. In contrast, for images low (-1 SD) in baseline intensity valence*repetition interaction was not significant (*t*(4000) = 0.05, *β* = -0.01, *p* = .96). There was, however, a significant main effect of time such that people’s emotional responses lost their intensity with repeated exposure (*t*(4000) = 2.73, *β* = -.28, *p* = 0.006) and did so at a similar rate for positive and negative images. The main effect of valence was not significant (*t*(4000) = 1.69, *β* = -.77, *p* = .09).

Finally, we examined the correlation between baseline affect ratings and the anxiety symptomatology composite. These correlations were computed after averaging the baseline affect ratings across images for each participant. For both negative and positive images, no significant correlations were found between anxiety symptomatology composite scores and average baseline affect (Negative: *r* = -0.05, *p* = 0.64; Positive: *r* = 0, *p* = 0.996).

**Part III - Additional Analyses on Image Themes for Study 1**

As reported in the main text, images were coded for: Excitement/Enthusiasm, Competition/Ambition, Romance/Love/Sensuality, Disgust/Revulsion, Loneliness/Isolation, Human Suffering (illness, pain), Death/Injury/Threat of physical harm, Male to Female Aggression, War/Terrorism, Outdoor activity/Sports, Sadness/Crying, Sensation seeking/adventure, Other. We examined the factorability between the themes and observed many correlations above 0.3. Bartlett’s test of sphericity was significant (χ2(66) = 231.93, p < .001) confirming the need for data reduction. We performed Exploratory Factor Analysis using varimax rotation to capture orthogonal latent variables (see Table S1.4 for details, and Table S1.5 for Pearson correlations between anxious symptomatology and the coded themes).


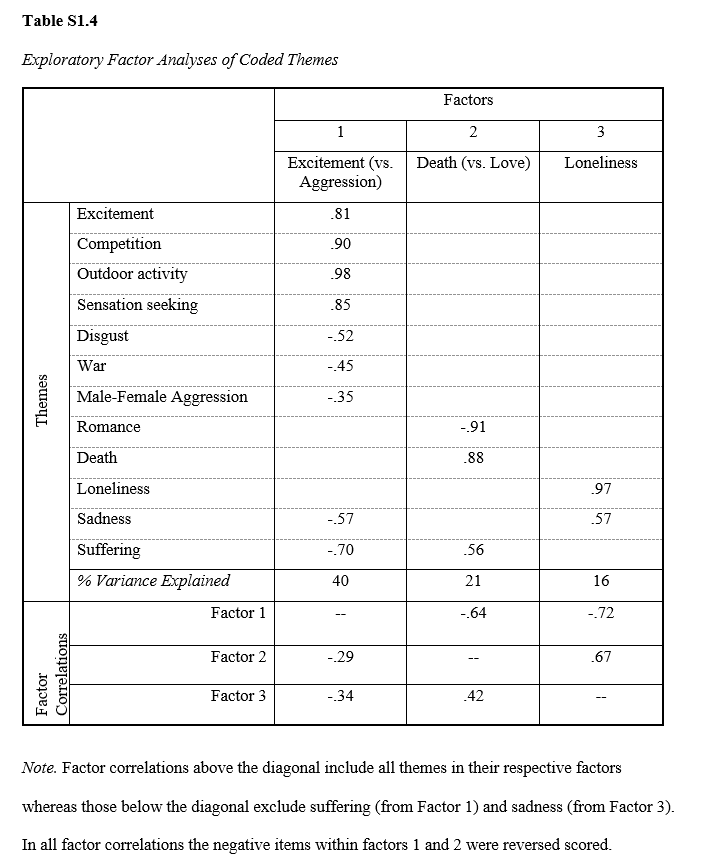


**Event Type (Interpersonal vs. Individual)**

In addition to thematic coding, images were also coded for whether or not they depicted scenes that were interpersonal/social. Any image that included more than one person were coded as interpersonal; images that included only a single person was coded as individual. For example, a scene describing a soldier pointing a gun at a child, or a scene of a romantic couple were coded as interpersonal, while images of a crying child or a lone skier were coded as individual. There were 11 interpersonal images (6 negative, 5 positive) and 9 individual images (4 negative, 5 positive). Because this coding simply required a count of people in each image, the first author did this coding on her own.

We ran logistic regression on event type (Interpersonal = 1, Individual = 0), with anxiety as the predictor. The results revealed no significant log ratio of anxiety scores in predicting interpersonal vs. individual events included in the images (*z* = -0.05, *b* = -.01, *p* = .96). Thus, the probability of viewing interpersonal vs. individual images during habituation was not dependent on anxiety scores.


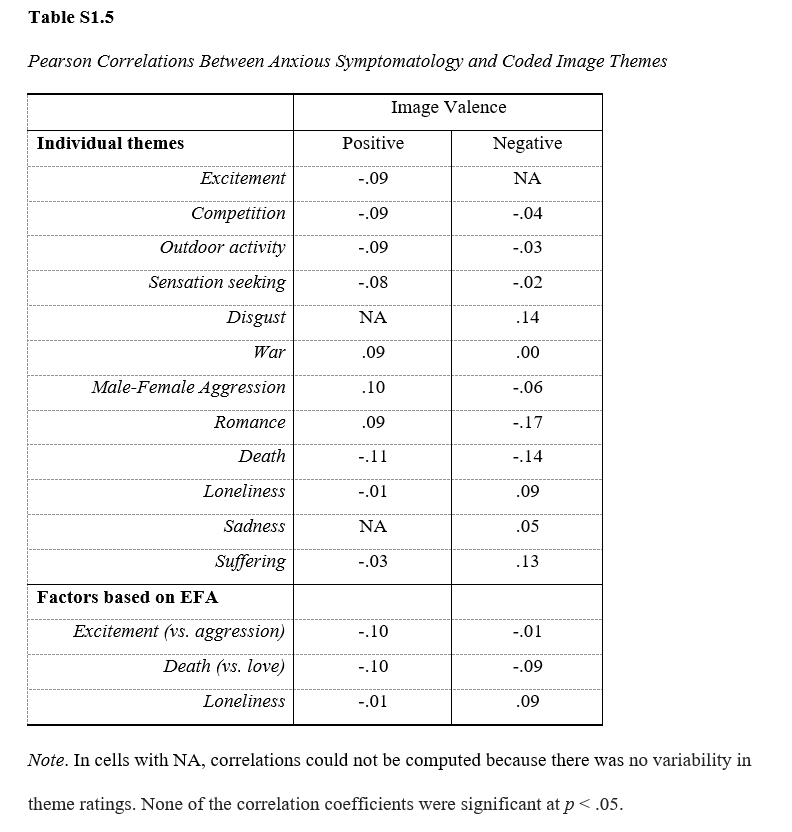


**Study 2**

Part I describes additional measures which were administered for exploratory purposes. Part II describes additional manipulation checks we performed on images themes and analyses on threat-challenge ratings. Part III includes analyses on additional moderators, including, interest, gender and image themes.

**Part I - Additional Measures for Study 2**

**Individual Differences Questionnaires**

The following measures were included in the survey part of Study 1:

- Big Five Inventory-2 (BFI2, Soto & John, 2017)
- Emotion Regulation Questionnaire (ERQ, Gross & John, 2003)
- Experiences in Close Relationships Scale – Short Form (ECR-short, Wei et al., 2007)
- The Temporal Distancing Questionnaire (Bruehlman-Senecal, Ayduk & John, 2016)
- The Emotion Reactivity Scale (PERS; Becerra et al., 2019)
- Responses to Positive Affect Questionnaire (RPA, Feldman, Joorman, & Johnson, 2008)
- Inventory of Responses to Positive Affective States (IRPAS, Wright & Armstrong, 2016)
- Savoring beliefs inventory (SBI, Bryant, 2003)
- Social Avoidance and Distress Scale (SADS; Watson & Friend, 1969)

**Image selection and image theme ratings**

***Habituation Stimuli Selection***

Selection of within-valence themes was based on Mikels et al (2005) and the supplementary analyses on emotion themes reported for Study 1 (see coded image themes above). Within negative and positive images sets, love and excitement images were matched on valence and arousal (*t*s < 1); so were fear and sadness images (valence: *t*(1.06) = 3.47, *p* = .17; arousal *t* < 1).

***Themes rating***

During the practice task of the second session, participants rated the 8 practice images on *Emotional theme* (How much does this image makes you feel each of the following specific emotions, right now, at this moment?). They were presented with the following emotional triplets: a. Inspired, uplifted (excited), or elevated; b. Love, closeness, or trust; c. Sad, downhearted, or unhappy; d. Scared, fearful, or afraid; e. Other. Each emotional triplet was rated on a scale from 1 (not at all) to 7 (very much so). Next, participants selected one *Dominant emotion theme* (“if you were to choose only one emotion this image made you feel the most, which one would you choose?”) from the following dropdown menu: a. Inspired, uplifted (excited), or elevated; b. Love, closeness, or trust; c. Sad, downhearted, or unhappy; d. Scared, fearful, or afraid (see Table S2.1 for descriptive statistics).

*
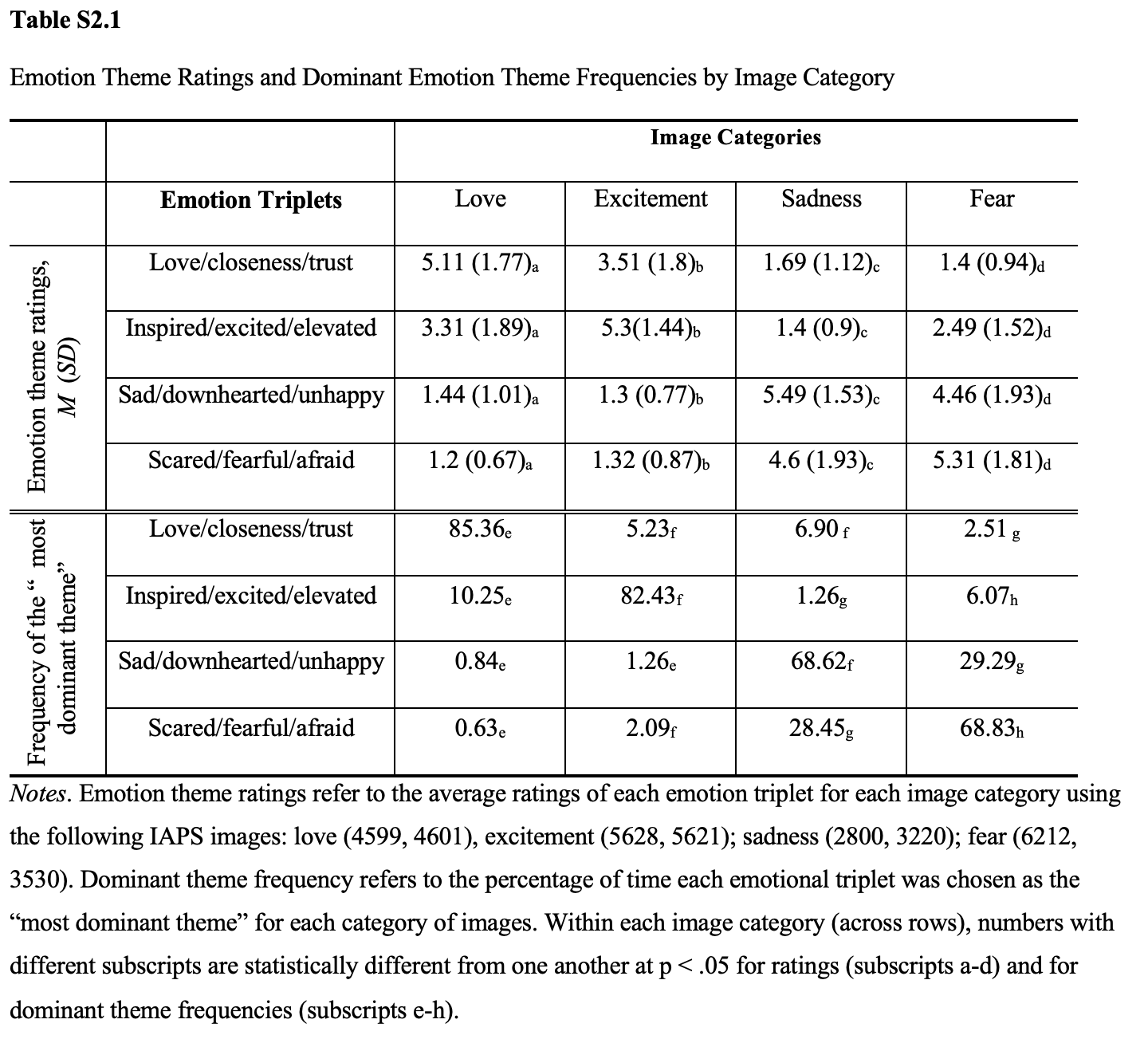
*

**State Emotions Throughout Session 2**

In addition to anxiety and interest ratings (described in main manuscript), participants were also asked to rate their level of excitement (How excited, enthusiastic, energized do you feel at this moment?), calmness (“How calm, relaxed, or at ease do you feel right now?) and sadness (“How sad, downhearted, or unhappy do you feel at this moment?”) using the same rating scale. These ratings were included as distractors and the data were not analyzed.

**Threat-Challenge Ratio**

As outlined in our pre-registration, following the Dream Job manipulation, we assessed threat-challenge ratings at three time points during the experimental procedure. Participants responded to two questions to assess demands (“How difficult and demanding do you find the upcoming Dream Job task?) and resources (“To what extent do you believe you have the ability and resources to do well on the Dream Job task?”). These questions were presented immediately after the manipulation, mid-habituation, and post-habituation. The threat-challenge index was calculated as the ratio of demands to resources (demands/resources), with the following index values at each time point: immediately post-manipulation (*M* = 1.29, *SD* = 1.18), mid-habituation (*M* = 1.42, *SD* = 1.40), and post-habituation (*M* = 1.55, *SD* = 1.48).

**Part II – Manipulation Check for Study 2**

**Image Themes**

To confirm our a priori categorization of the images to 4 emotion themes, we examined participants’ ratings of excitement, love, sadness and fear for each image category. Excitement images were rated significantly higher on excitement than on love (*t*(910.79) = 18.50, *p* <.001), sadness (*t*(817.87) = 50.54, *p* <.001) and fear (*t*(971.62) = 29.668, *p* <.001). Love images were rated significantly higher on love than on excitement (*t*(973.77) = 13.93, *p* <.001), fear (*t*(739.54) = 40.84, *p* <.001) and sadness (*t*(824.65) = 35.94, *p* <.001). Similarly, sadness images were higher on sadness ratings than on fear (*t*(942.45) = 9.24, *p* <.001), excitement (*t*(727.88) = 54.49, *p* <.001) and love (*t*(857.49) = 49.12, *p* <.001) ratings. Lastly, fear images were rated higher on fear compared to sadness (*t*(966.18) = 5.87, *p* <.001), excitement (*t*(695.05) = 43.78, *p* <.001) and love (*t*(616.01) = 46.79, *p* <.001).

Analyses of responses to the dominant emotional theme confirmed that “love, closeness or trust” triplet was the most frequent theme chosen for love images, “inspired, uplifted, or elevated” for excitement images, “sad, downhearted, or unhappy” for sadness images, and “scared, fearful, or afraid” for fear images. Although fear and sadness images were less well-differentiated from each other than love vs. excitement images, fear and sadness triplets were nevertheless chosen significantly more as the dominant theme for fear and sadness images, respectively.

**Threat-Challenge Ratio**

Consistent with our pre-registered expectations, the threat-challenge ratios were found to be higher in the experimental group immediately following manipulation (*M_E_* = 1.46 , *SD_E_* = 1.39; *M_C_* = 1.08, *SD_C_* = 0.79; *t*(68191) = 47.8, *p* < .001), and the differences maintained throughout the habituation task (mid habituation: *M_E_* = 1.62, *SD_E_* = 1.66 ; *M_C_* = 1.19, *SD_C_* = 0.93 ; *t*(67144) = 45.44, *p* < .001; post habituation: *M_E_* = 1.72, *SD_E_* = 1.67; *M_C_* = 1.35 , *SD_C_* = 1.16; *t*(73979) = 35.76, *p* < .001). Additionally, threat-challenge ratings significantly increased in both groups from post-manipulation to mid-habituation (*t_E_* (80471) = -14.71, *p* < .001;*t*_C_ (66564) = -15.99, *p* < .001) and from mid-habituation to post-habituation (*t*_E_ (83119) = -8.19, *p* < .001; *t*_C_ (65153) = -20.01, *p* < .001).

Group differences were significant, with the experimental group showing a larger increase in threat-challenge ratings from post-manipulation to mid-habituation (*t_diff_* (74585) = 3.46, p < .001), and smaller increase in threat challenge-ratings from mid-habituation to post habituation (*t_diff_* (59705) = -5.12, p < .001).

**Part III – Additional Moderator Analyses for Study 2**

**Interest**

Interest ratings declined across time during the habituation task as presented in Figure S2.1. However, interest levels were not significantly different between groups at baseline (*t*(457.48) = -0.03, *p* = .98) nor the mean ratings during the habituation phase were different between groups, *t*(13.27) = -0.42, *p* = .68.


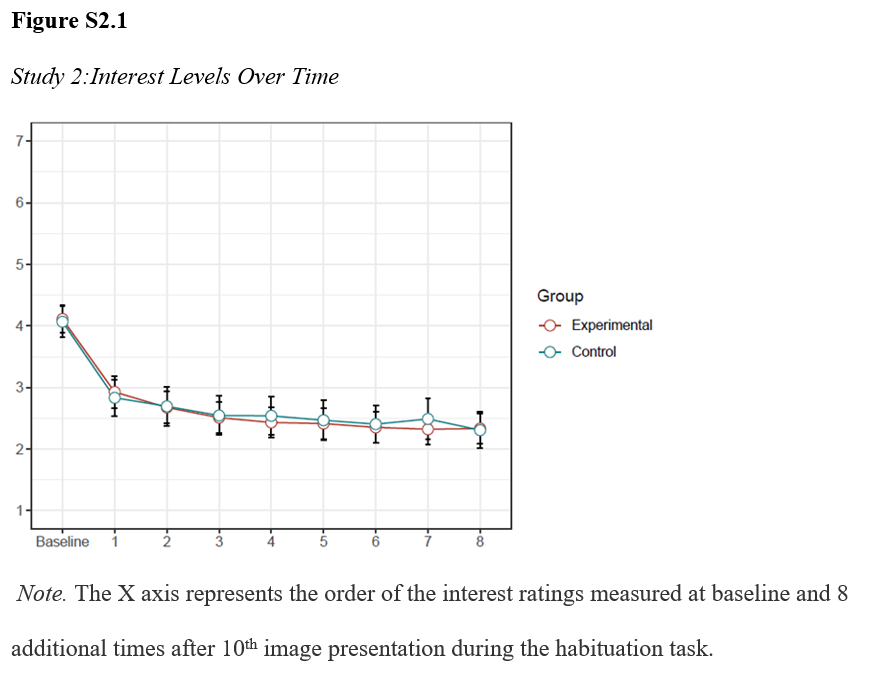


To examine the change in interest ratings during the habituation task, we predicted interest ratings from the interest rating order within the habituation task (1-8, fixed and random effects). Results indicated that interest ratings declined significantly across images during habituation task, *t*(1624) = 6.63, *β* = -0.08, *p* <.001. However, this decline in interest over time was not moderated by valence, since adding valence (2: negative vs. positive, fixed and random effects) to the model did not result in a 2-way interaction between image order*valence, *t*(1622) = -0.14, *β* = -0.01, *p* = .89.

**Gender**

We examined whether habituation might vary as function of individual differences in anxiety and gender. We included gender as an additional moderator in a mixed effect model, predicting affect from valence (2: negative vs. positive, fixed and random effects), repetition (continuous, fixed and random effects), post-manipulation anxiety (continuous, fixed effects), and gender (2: male vs. female, fixed effects). The 4-way interaction between valence*repetition*post-manipulation anxiety*gender was not significant (*t*(18869) = 0.04, *β* = -0.00, *p* = .97).

**Baseline Affect Intensity**

Baseline affect was grand-mean centered and included as a fixed effect in the base model, which examined valence and repetition as predictors of emotional responses. The three-way interaction was not significant, t(18,874) = 0.53, β = -0.002, p = 0.60. However, the Repetition × Valence interaction remained significant, t(18,874) = 9.51, β = -0.59, p < .001.

In addition, we examined the correlation between baseline affect and anxiety symptomatology composite. These correlations were computed after averaging the baseline affect ratings across images for each participant. For negative image trials, the correlation was small and not significant (r = -0.06, p = 0.36), whereas for positive image trials, anxiety symptomatology was negatively correlated with baseline affect (r = -0.15, p = 0.02).

**Replicating Study 1 Findings on Anxiety Symptomatology and Valence Differences in the Full Sample**

To explore whether the three-way interaction between repetition, valence, and anxiety symptomatology observed in Study 1 could be conceptually replicated in Study 2, we tested this interaction using repetition (continuous, 1-10; fixed), valence (positive vs. negative; fixed), and anxiety symptomatology in relation to affect ratings. In the main manuscript, we focused on the control group, as its design is more comparable to Study 1 (as specified in our preregistration [#73120](https://aspredicted.org/3qa57.pdf)). However, we would also like to share the results from analyses conducted on the full sample and on the experimental group.

When we examined the entire sample, the three-way interaction between repetition, valence, and anxiety symptomatology was not significant (*t*(18875) = -1.56, *b* = -0.10, *p* = 0.12), and the same was true for the experimental group only (*t*(10343) = -0.59, *b* = -0.05, *p* = 0.55). However, the marginal effect that was observed in the control group (*t*(8526) = 1.87, *b* = -0.18, *p* = 0.06), is similar to the significant effect seen in Study 1. These findings support our view, since the experimental group demonstrated behavior more closely aligned with the original study.

**Potential Moderation by Image Theme**

Differently from Study 1, Study 2 included fixed stimuli by emotional theme (sadness vs fear; love vs excitement). Having fixed stimuli between participants allowed us to systematically examine whether thematic differences in emotion elicitors resulted in different habituation trajectories. Among negative stimuli, we predicted affect from repetition (continuous, 1-10; fixed and person centered on time 1), emotion elicitor theme (effect coded: fear = 1, sadness = -1), repetition*emotion elicitor, with random intercepts and slopes. Consistent with previous findings, results showed a main effect for repetition, t(9318) = 5.22, b = -0.29, p <.001, with decreasing affect over time, however the two-way interaction between repetition and emotion elicitor type was not significant, t(9318) = 1.22, b = 0.08, p = 0.22, indicating no substantial differences between sadness and fear in habituation rates over time.

We conducted parallel analyses for positive elicitors, predicting affect intensity from repetition (continuous, 1-10; fixed and person centered on time 1), emotion elicitor (effect coded: love = 1, excitement = -1), repetition*emotion elicitor, with random intercepts and slopes. We found a main effect for repetition (t(9318) = 5.43, b = -0.34, p < .001) with decreasing affect intensity over time, and again the two-way interaction between repetition and emotion elicitor type was not significant (t(9318) = 0.65, b = -0.05, p = 0.52), indicating no substantial differences in habituation rates between love and excitement.

**Comparison of Anxiety Symptomatology Across Studies in the Context of COVID-19 Pandemic**

To investigate the potential influence of increased anxiety levels in the general population due to the COVID-19 pandemic, we compared anxiety symptomatology between Study 1 and Study 2. Our analyses revealed no statistically significant difference in raw anxiety composite scores between the two studies (*t*(249.03) = 0.93, *p* = 0.35; Study 1: *M* = 11.41, *SD* = 7.81, *α* = 0.83; Study 2: *M* = 12.34, *SD* = 10.05, *α* = 0.84). These results suggest that anxiety levels were comparable across both samples.

**References**

Becerra, R., Preece, D., Campitelli, G., & Scott-Pillow, G. (2019). The assessment of emotional reactivity across negative and positive emotions: Development and validation of the Perth Emotional Reactivity Scale (PERS). *Assessment, 26*(5), 867-879.

Bruehlman-Senecal, E., Ayduk, Ö., & John, O. P. (2016). Taking the long view: Implications of individual differences in temporal distancing for affect, stress reactivity, and well-being.*Journal of Personality and Social Psychology, 111*(4), 610-635.

Bryant, F. B. (2003). Savoring beliefs inventory (SBI): A scale for measuring beliefs about savouring. *Journal of Mental Health, 12*(2), 175–196.

Carver, C. S., & White, T. L. (1994). Behavioral inhibition, behavioral activation, and affective responses to impending reward and punishment: the BIS/BAS scales. *Journal of Personality and Social Psychology*, *67*(2), 319-333.

Downey, G., & Feldman, S. I. (1996). Implications of rejection sensitivity for intimate relationships. *Journal of Personality and Social Psychology*, *70*(6), 1327-1343.

Feldman, G. C., Joormann, J., & Johnson, S. L. (2008). Responses to positive affect: A self-report measure of rumination and dampening. *Cognitive Therapy and Research*, *32*(4), 507-525.

Gross, J. J., & John, O. P. (2003). Individual differences in two emotion regulation processes: implications for affect, relationships, and well-being. *Journal of Personality and Social Psychology*, *85*(2), 348-362.

John, O. P., Donahue, E. M., & Kentle, R. L. (1991). Big five inventory. *Journal of Personality and Social Psychology*.

Lovibond, P. F., & Lovibond, S. H. (1995). The structure of negative emotional states: Comparison of the Depression Anxiety Stress Scales (DASS) with the Beck Depression and Anxiety Inventories. *Behaviour Research and Therapy*, *33*(3), 335-343.

Mikels, J. A., Fredrickson, B. L., Larkin, G. R., Lindberg, C. M., Maglio, S. J., & Reuter-Lorenz, P. A. (2005). Emotional category data on images from the International Affective Picture System. *Behavior Research Methods*, *37*(4), 626-630.

Radloff, L. S. (1977). The CES-D scale: A self-report depression scale for research in the general population. *Applied Psychological Measurement*, *1*(3), 385-401.

Soto, C. J., & John, O. P. (2017). The next Big Five Inventory (BFI-2): Developing and assessing a hierarchical model with 15 facets to enhance bandwidth, fidelity, and predictive power. *Journal of Personality and Social Psychology*, *113*(1), 117.

Treynor, W., Gonzalez, R., & Nolen-Hoeksema, S. (2003). Rumination reconsidered: A psychometric analysis. *Cognitive Therapy and Research*, *27*(3), 247-259.

Watson, D., & Friend, R. (1969). Measurement of social-evaluative anxiety. Journal of Consulting and Clinical Psychology; *Journal of Consulting and Clinical Psychology*, *33*(4), 448.

Wei, M., Russell, D. W., Mallinckrodt, B., & Vogel, D. L. (2007). The experiences in Close Relationship Scale (ECR)-Short Form: Reliability, validity, and factor structure. *Journal of Personality Assessment*, *88*, 187-204.

Wright, K., & Armstrong, T. (2016). The Construction of an Inventory of Responses to Positive Affective States. *SAGE Open*, *6*(1).
